# Dynapenic Abdominal Obesity as a Risk Factor for Worse Trajectories of ADL Disability Among Older Adults: The ELSA Cohort Study

**DOI:** 10.1093/gerona/gly182

**Published:** 2018-08-25

**Authors:** Tiago da Silva Alexandre, Shaun Scholes, Jair Licio Ferreira Santos, Cesar de Oliveira

**Affiliations:** 1 Department of Epidemiology and Public Health, University College London, UK; 2 Department of Gerontology, Federal University of Sao Carlos; 3 Department of Social Medicine, University of Sao Paulo, Ribeirao Preto, Brazil

**Keywords:** Handgrip, Dynapenia, Weakness, Waist circumference, Disability

## Abstract

**Background:**

There is little epidemiological evidence demonstrating that dynapenic abdominal obese individuals have worse trajectories of disability than those with dynapenia and abdominal obesity alone. Our aim was to investigate whether dynapenic abdominal obesity can result in worse trajectories of activities of daily living (ADL) over 8 years of follow-up.

**Methods:**

We used longitudinal data from 3,723 participants free from ADL disability at baseline from the English Longitudinal Study of Ageing. Using measures of handgrip strength (<26 kg for men; <16 kg for women) and waist circumference (>102 cm for men; >88 cm for women), participants were classified into four groups: nondynapenic/nonabdominal obese (reference group), abdominal obese only, dynapenic only, and dynapenic abdominal obese. We used generalized linear mixed models with ADL as the outcome and the four groups according to dynapenia and abdominal obesity status as the main exposure controlled by sociodemographic, behavioral, and clinical characteristics.

**Results:**

The estimated change over time in ADL disability was significantly higher for participants with dynapenic abdominal obesity compared with those with neither condition (+0.018, 95% CI: 0.008 to 0.027). Compared with the results of our main analysis (which took into account the combination of dynapenia and abdominal obesity on the rate of change in ADL), the results of our sensitivity analysis—which examined dynapenia and abdominal obesity only as independent conditions—showed an overestimation of the associations of dynapenia only and of abdominal obesity only on the ADL disability trajectories.

**Conclusions:**

Dynapenic abdominal obesity is an important risk factor for functional decline in older adults.

Aging is associated with changes in body composition, characterized by an increase in the percentage of body fat and a decrease in lean mass (1). With regards to body fat distribution, aging is associated with an increase in central adiposity and an increase in fat deposition in muscle, along with a reduction in subcutaneous fat (2,3). Recent evidence has shown that fatty infiltration of muscle is an important component of low muscle strength and that abdominal obesity can reduce muscle strength through inflammatory and endocrine mechanisms (2–5).

Schaap and colleagues (6) in a recent meta-analysis showed that obesity (body mass index [BMI] ≥30 kg/m^2^) and poor muscle strength (dynapenia) were associated with functional decline over time, but no such association was found for muscle mass. They also found that waist circumference (WC) was strongly associated with mobility disability and with activities of daily living (ADL) disability (5,7–10).

Previous studies have analyzed obesity and dynapenia as two independent conditions (11). However, a limitation of this approach is that a dynapenic abdominal obese individual can be classified as either dynapenic only or abdominal obese only resulting in an overestimation of the associations of dynapenia only and abdominal obesity only with conditions such as disability.

Although five longitudinal studies have examined the combination of obesity and dynapenia in their analyses of disability and mobility limitation (12–16), only two studies had ADL as outcome (12,15). However, these two studies have not analyzed such associations using trajectory models over time.

Given the lack of epidemiological evidence showing dynapenic abdominal obesity as a risk factor for the progression of ADL disability, the aim of the present study was to investigate whether dynapenic abdominal obesity was associated with trajectories of increasing ADL disability among English older adults over 8 years of follow-up. We tested the following hypotheses: (a) trajectories of ADL disability are worse in dynapenic abdominal obese individuals compared with those nondynapenic/nonabdominal obese individuals and (b) choosing an analytical strategy that does not consider dynapenic abdominal obesity as a condition (ie, treats dynapenia and abdominal obesity as separate conditions) overestimates the associations between dynapenia and abdominal obesity and the trajectories of ADL disability.

## Methods

Data were extracted from English Longitudinal Study of Ageing (ELSA) that is a panel study that began in 2002 with a representative cohort of men and women aged 50 and older living in England. The ELSA sample comprised 11,391 individuals who had previously participated in the Health Survey for England, an annual health examination survey, which each year recruits a different nationally representative sample using a multistage stratified random probability design. After the baseline year, follow-up interviews within ELSA occur every 2 years and health examinations, that is, a nurse visit, every 4 years. Detailed descriptions of the study design and the sampling procedures have been previously published (17).

We included participants aged 60 years or older in 2004, when anthropometric data were collected for the first time. Overall, 6,180 ELSA participants had valid data on ADL in 2004. Of these, 1,532 were excluded from our analytical sample due to participants reporting at least one disability in ADL. In addition, a further 886 persons were excluded due to missing data on handgrip strength, WC, or other covariates, and a further 39 were excluded due to being underweight (BMI < 18.5 kg/m^2^), resulting in a final analytical sample of 3,723 individuals. Participants who were underweight were excluded from the analytical sample in order to avoid bias in our results because underweight is an important risk factor for ADL limitation (18).

The participants were reassessed at 4 and 8 years of follow-up. All ELSA participants gave written informed consent. The National Research and Ethics Committee granted ethical approval for all the ELSA waves (http://www.nres.npsa.nhs.uk/) (MREC/01/2/91).

### ADL Disability

Data on self-reported ADL were collected at baseline and at each follow-up visit. Disability was defined herein as a difficulty to perform the following ADL: walking, transferring, toileting, bathing, dressing or feeding, according to the modified Katz Index (19,20). Despite its importance regarding functionality among elderly individuals, incontinence was not included because it does not necessarily imply physical limitation (21). The six ADL items were summed to form a scale that ranged from 0 to 6, with 0 representing no disability in ADL. Only individuals without any ADL disability at baseline were included in our analysis.

### Anthropometric Measurements and Classification of the Groups

A trained evaluator carried out the WC measurement with a flexible tape placed at the midpoint between the last rib and the iliac crest. The participants remained upright with the arms alongside the body, without the upper portion of their clothes, and were instructed to relax the abdomen. The WC measurement was taken at the end of the expiratory phase of a breathing cycle. Abdominal obesity was defined by WC >102 cm for men and by >88 cm for women (22).

Grip strength was measured three times for each hand using the Smedley dynamometer. Maximum strength tests were performed with a 1-minute rest between tests, and the highest strength value in the dominant hand was used in our analysis. Dynapenia was defined based on two cutoff points for grip strength: less than 26 kg for men and less than 16 kg for women, in accordance with the recommendations of the Foundation for the National Institutes of Health Biomarkers Consortium Sarcopenia Project (23).

At each visit, a four-category variable was created based on participants’ dynapenia and abdominal obesity status. The categories were as follows: nondynapenic/nonabdominal obese, abdominal obese only, dynapenic only, and dynapenic abdominal obese. This was used in our analysis as a time-varying covariate.

BMI values for participants were calculated by dividing weight in kilograms by height in meters squared (kg/m^2^). This was used in our analysis as a continuous variable.

### Covariates

Sociodemographic characteristics included age, sex, marital status, income, and educational level. Age was grouped into three 10-year categories, with the participants aged 80 years or older combined into one group. Marital status was classified as married (married individuals or those in a stable relationship) and as not married (divorced, separated, or widowed individuals). Household wealth in quintiles was used as a measure of socioeconomic status. Educational status was grouped into three categories: lower than “O-level” or equivalent (0–11 years of schooling), qualified to a level lower than “A-level” or equivalent (12–13 years), and a higher qualification (>13 years).

Smoking status was assessed by asking participants whether they were a nonsmoker, former smoker, or a current smoker. Frequency of alcohol consumption was classified as nondrinkers or drinking on 1 day a week, drinking on 2 to 6 days a week (frequently), or drinking daily. Self-reported physical activity data were collected using three questions on the frequency of participation in vigorous-, moderate-, and mild-intensity physical activities, with the response options for each being more than once per week, once per week, one to three times per month, or hardly ever. Physical activity was further categorized into the following two groups: sedentary lifestyle (no activity on a weekly basis) or active (mild, moderate, or vigorous activity at least once a week).

Systemic arterial hypertension, diabetes, cancer, lung disease, heart disease, stroke, osteoarthritis, and falls were recorded based on self-reports. The presence of depressive symptoms was defined using the Center for Epidemiologic Studies Depression Scale (CESD) score (CESD ≥ 4).

Cognitive function was assessed using tests of immediate and delayed verbal memory. This consisted of presenting to participants a list of 10 nouns aurally on a computer, one noun every 2 seconds. Participants were asked to recall as many of the 10 words as possible immediately, and again after a short delay, during which they carried out other cognitive tests. We computed an overall memory score (range 0–20) using both the immediate and delayed recall results. Perception of hearing (response options: good/regular/poor) and perceptions of near and far vision (response options: good/regular/poor) were also included in our analysis.

All the covariates included in our analyses represented a wide range of risk factors associated with the progression of ADL disability (20). All variables were treated in our analyses as time-varying covariates, with the exception of age, sex, and level of education.

### Statistical Analyses

Descriptive data at baseline were expressed as means, standard deviations, and as percentages. Differences in baseline characteristics between (a) included and excluded individuals from the analytical samples due to missing data on handgrip strength, WC, or other covariates and (b) the four analytical groups classified on the basis of participants’ dynapenia and abdominal obesity status were assessed using the chi-square test, analysis of variance, and by post hoc Tukey tests. For all analyses, *p* <.05 was used to indicate statistical significance.

To estimate the trajectories in ADL disability, we used generalized linear mixed models using the XTMIXED procedure in Stata 14 SE program (Stata Corp, College Station, TX). These models were chosen because they best handle unbalanced data from studies with repeated measures and they enable the statistical modeling of changes in the time-dependent outcome variable (ADL score), as well as allowing time-dependent change in the magnitude of associations between variables (24,25).

As all participants were free from ADL disability at the baseline visit, the estimates from the mixed models represent the estimated change in ADL score over a follow-up period of 1 year (ie, a one-unit increase in time).

We entered a time by dynapenia/abdominal obesity status interaction term into our models to estimate the difference in the change in ADL score for a one-unit increase in time between the dynapenic abdominal obese group and the reference group (neither dynapenia nor abdominal obesity). Similar comparisons to the reference group were made for the dynapenia only and for the abdominal obesity only groups. The interaction terms therefore enable the pace of change in the ADL score to vary according to the four dynapenia/abdominal obesity groups.

Univariate analyses were run to choose the most optimal set of covariates to adjust for in the final models. Only those covariates showing associations with a *p* value ≤.20 in univariate analyses were selected for inclusion in the multivariate models, in which forward stepwise selection was used to find the optimal set of covariates.

We performed a sensitivity analysis to investigate our second hypothesis in which dynapenia only (yes/no) and abdominal obesity only (yes/no) were analyzed in the models of ADL trajectories.

## Results

Of the 3,723 participants at baseline with no ADL disability, 2,812 and 2,360 were reassessed at 4 and 8 years of follow-up, respectively. Just more than 60.4% of the analytical sample (*n* = 2,247) took part in all three waves, 18.2% took part in two waves (*n* = 678), and 21.4% took part in just the baseline visit (*n* = 798).

Baseline characteristics of all participants according to the four dynapenia/abdominal obesity groups are shown in Table 1. Differences in baseline characteristics between included and excluded individuals are shown in Supplementary Table 1.

**Table 1.** Baseline Characteristics of 3,723 Older Adults From the ELSA Study (2004) According to Abdominal Obesity and Dynapenia Status

	Nondynapenic/Nonabdominal Obese	Abdominal Obese	Dynapenic	Dynapenic Abdominal Obese	Total Sample
	*n* = 1,732	*n* = 1,668	*n* = 174	*n* = 149	*n* = 3,723
Sociodemographic variables					
Age, y (*SD*)	71.3 ± 7.2	71.5 ± 6.9	79.7 ± 8.7*^,†^	77.4 ± 9.6*^,†,‡^	72.0 ± 7.5
60–69 years old	58.3	54.9*	18.4*^,†^	31.5*^,†,‡^	53.8
70–79 years old	31.8	35.8*	39.1*^,†^	31.6*^,†,‡^	33.9
80 or more years old	9.9	9.3*	42.5*^,†^	36.9*^,†,‡^	12.3
Sex (female, %)	48.0	59.1*	56.3*	63.1*	54.0
Marital status (married, %)	69.7	67.9	45.4*^,†^	49.7*^,†^	67.0
Household wealth, (%)					
Fifth quintile (highest quintile)	10.9	15.5*	25.9*^,†^	24.8*^,†^	24.3
Fourth quintile	15.8	19.8*	23.5*^,†^	28.2*^,†^	21.4
Third quintile	19.9	21.3*	16.1*^,†^	18.1*^,†^	20.1
Second quintile	23.1	20.5*	20.1*^,†^	18.1*^,†^	18.3
First quintile (lowest quintile)	29.0	21.8*	14.4*^,†^	10.1*^,†^	15.3
Unreported	1.3	1.1*	0.0	0.7*	0.6
Schooling					
Higher than A level, (%)	27.8	21.5*	15.6*^,†^	9.4*^,†^	23.6
O-level or equivalent, (%)	23.8	20.8*	14.9*^,†^	19.5*^,†^	21.9
Less than O-level or equivalent, (%)	48.4	57.7*	69.5*^,†^	71.1*^,†^	54.5
Behavioral variables					
Smoking					
Nonsmoker, (%)	39.8	36.6*	31.0*	31.5*	37.6
Former smoker, (%)	47.8	52.2*	58.1*	58.4*	50.7
Current smoker, (%)	12.4	11.2*	10.9*	10.1*	11.7
Alcohol intake, (%)					
Nondrinkers or drank once a week	30.6	38.2*	35.6*^,†^	43.6*^,†^	34.8
Drank frequently, (%)	42.9	36.8*	32.2*^,†^	29.6*^,†^	39.1
Drank daily, (%)	19.1	16.2*	14.4*^,†^	8.7*^,†^	17.2
Did not answer, (%)	7.4	8.8*	17.8*^,†^	18.1*^,†^	8.9
Sedentary lifestyle, (%)	2.1	2.5	8.0*^,†^	5.4*^,†^	2.7
Clinical conditions					
Arterial hypertension (yes; %)	15.3	21.5*	19.5	21.5*	18.5
Diabetes (yes; %)	2.1	4.6*	3.4	5.4*	3.4
Cancer (yes; %)	2.7	4.8*	3.5	2.0	3.7
Lung disease (yes; %)	10.8	13.7*	12.6	16.8*	12.4
Heart disease (yes; %)	7.7	9.9*	10.9	14.1*	9.1
Stroke (yes; %)	1.5	0.8	0.6	2.7^†^	1.2
Osteoarthritis, (%)	24.0	34.6*	51.5*^,†^	61.7*^,†^	31.5
Falls (yes; %)	24.9	27.2	35.1*^,†^	36.2*^,†^	26.9
Mean memory score, points (*SD*)	9.9 ± 3.3	9.8 ± 3.3	8.0 ± 3.9*^,†^	7.9 ± 3.6*^,†^	9.7 ± 3.4
Depression, (%)	8.0	11.6*	17.8*^,†^	14.1*	10.3
Perception of hearing, (%)					
Good	78.4	79.9	74.7	74.5	78.8
Regular	17.1	16.0	19.0	22.8	16.9
Poor	4.5	4.1	6.3	2.7	4.3
Perception of vision, (%)					
Good	90.1	89.9	79.9*^,†^	81.2*^,†^	89.2
Regular	8.2	7.8	14.9*^,†^	14.8*^,†^	8.6
Poor	1.7	2.3	5.2*^,†^	4.0*^,†^	2.2
Handgrip strength, kg (*SD*)	32.2 ± 9.8	31.0 ± 9.9*	15.6 ± 5.9*^,†^	15.4 ± 5.5*^,†^	30.2 ± 10.6
Waist circumference, cm (*SD*)	87.3 ± 8.7	102.9 ± 9.4*	85.0 ± 8.7*^,†^	102.4 ± 10.0*^,‡^	94.8 ± 12.0
Body mass index, kg/m^2^ (*SD*)	24.8 ± 2.5	30.3 ± 3.8*	23.9 ± 2.6*^,†^	30.0 ± 4.2*^,‡^	27.4 ± 4.3

Notes: Data are presented as percentages, means, and standard deviation. Wealth cut-points values: highest quintile = more than £423 k; fourth quintile = between £240 and £423 k; third quintile = between £137 and £240 k; second quintile = between £24 and £137 k; lowest quintile = less than £24 k. Abbreviations: ELSA = English Longitudinal Study of Ageing; *SD* = standard deviation.

*Significantly different from nondynapenic/nonabdominal obese;

^†^Significantly different from abdominal obese;

^‡^Significantly different from dynapenic.

Statistical significance was set as *p* < .05.

The prevalence of dynapenic abdominal obesity, dynapenia only, and abdominal obesity only at baseline was respectively 4.0% (95% CI: 3.4 to 4.7), 4.7% (95% CI: 4.0 to 5.4), and 44.8% (95% CI: 43.2 to 46.4).

Table 2 presents the results from generalized linear mixed models that estimated associations between dynapenia and abdominal obesity status and ADL disability trajectories. The estimated change over time in the ADL score was stable for the reference group (when all other covariates in the model were at zero or at average values). In other words, according to the estimated coefficient (slope = 0.004; 95% CI: −0.007 to 0.015), there was no significant decline in the estimated ADL score for a one-unit increase in time for the following individuals: aged 60–69 years, male, those who remained nondynapenic/nonabdominal obese, did not report a sedentary lifestyle, perceived their vision as good, had a CESD score <4 points, did not report osteoarthritis, stroke and lung disease, reported no falls, had a mean memory score of 20, and were in the highest wealth quintile.

**Table 2.** GLM Estimates for ADL Score as a Function of Dynapenia and Abdominal Obesity Status Over a 8-Year Period in English Older Adults (*N* = 3,723)

Parameter	Estimate	Lower 95% CI to Upper 95% CI
Time, y	0.004	(−0.007 to 0.015)
Nondynapenic/nonabdominal obesity	Reference	
Time × Abdominal Obesity	0.004	(−0.002 to 0.010)
Time × Dynapenia	−0.001	(−0.010 to 0.009)
Time × Dynapenia/Abdominal Obesity	0.018	(0.008 to 0.027)**

Notes: There is no term to represent the difference in the estimated ADL score at baseline as all participants had no ADL disability. Model adjusted by age, sex, sedentary lifestyle, perception of vision, depressive symptoms, osteoarthritis, stroke, lung disease, falls, mean memory score, and wealth. Abbreviations: ADL = activities of daily living; CI = confidence interval; GLM = generalized linear mixed models.

**p < .001.

Compared with nondynapenic nonabdominal obese participants (the reference group), the estimated increase in ADL score for a one-unit increase in time was higher for dynapenic abdominal obese participants. The parameter estimate for the difference in slope between the two groups was +0.018 points per year (95% CI: 0.008 to 0.027) after adjusting for sex, age, sedentary lifestyle, perception of vision, depression, osteoarthritis, stroke, lung disease, memory score, and household wealth quintile (Table 2).

Compared with the results of our main analysis (which took into account the combination of dynapenia and abdominal obesity on the rate of change in ADL), the results of our sensitivity analysis—which examined dynapenia and abdominal obesity only as independent conditions—showed an overestimation of the associations of dynapenia only and of abdominal obesity only on the ADL disability trajectories (Table 3). That is, the terms representing dynapenia and abdominal obesity as separate conditions only attained statistical significance after removing from the model the term representing the combined conditions.

**Table 3.** GLM Estimates for ADL Score as a Function of Dynapenia and Abdominal Obesity Only Over a 8-Year Period in English Older Adults (*N* = 3,723)—Sensitivity Analysis

Parameter	Estimate	Lower 95% CI to Upper 95% CI
Time, y	0.003	(−0.008 to 0.014)
Time × Abdominal Obesity (yes)	0.006	(0.001 to 0.012)*
Time × Dynapenia (yes)	0.007	(0.001 to 0.014)*

Notes: There is no term to represent the difference in the estimated ADL score at baseline as all participants had no ADL disability. Model adjusted by age, sex, sedentary lifestyle, perception of vision, depressive symptoms, osteoarthritis, stroke, lung disease, falls, mean memory score, and wealth. Abbreviations: ADL = activities of daily living; CI = confidence interval; GLM = generalized linear mixed models.

*p < .05.

**Figure 1.**
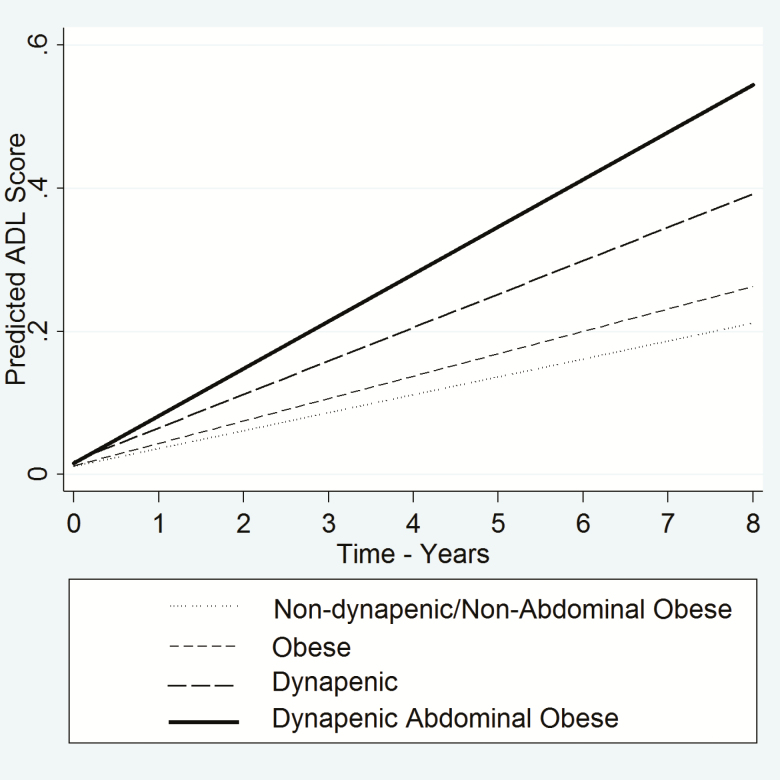
Trajectories of ADL disability according to dynapenia and abdominal obesity status—ELSA study 2004–2012. Abbreviations: ADL = activities of daily living; ELSA = English Longitudinal Study of Ageing.

The estimated trajectories in ADL disability among participants with dynapenic abdominal obesity increased more rapidly over the follow-up period compared with those with neither condition (Figure 1). The rates of change in ADL disability for participants with dynapenia only and with abdominal obesity only were similar to those with neither condition.

The average annual increases of ADL change for the four groups were nondynapenic/nonabdominal obese = 0.026 per year; abdominal obese only = 0.030 per year; dynapenic only = 0.025 per year; dynapenic/abdominal obese = 0.043 per year (*p* < .05 compared with nondynapenic/nonabdominal obese participants).

## Discussion

Our main findings showed that dynapenic abdominal obese participants presented an annual average increase in ADL disability, that is, difference in the rate of ADL change, almost twofold compared with those with neither condition. Dynapenia only and abdominal obesity only participants had trajectories in ADL disability that were similar to participants with neither condition. These findings draw attention to the combined effect of central fat distribution and simultaneous muscle weakness on incident disability later in life. The easy identification by health professionals of older adults with both conditions could help prevent worse trajectories of loss in functional ability in this group.

Rossi and colleagues (12) found that dynapenic abdominal obesity was associated with worsening disability during 10 years of follow-up. However, the authors used Cox regression models and included in the sample individuals with disability at baseline, limiting comparability with our findings. In addition, the decision by Rossi and colleagues (12) to exclude individuals unable to walk at least half a mile, with cognitive decline, renal failure, disabling knee osteoarthritis, heart failure, cancer, and serious lung disease, that is, the exclusion of persons with many of the known major risk factors for disability, may have allowed them to find statistical associations between dynapenia only and abdominal obesity only with disability. In our study, retaining participants with these risk factors in the analytical samples, we found that dynapenia only and abdominal obesity only participants had trajectories in ADL disability that were similar to those observed among participants with neither condition. Furthermore, our sensitivity analysis showed that the use of statistical models which did not take the combination of dynapenia and abdominal obesity into account could lead to an overestimate of the association between dynapenia only and abdominal obesity only on the change over time in ADL disability.

In another study, Stenholm and colleagues (13), analyzing 930 individuals aged 65 years and over followed over a 6-year period, using BMI and knee extensor strength as the key exposure measures, found that obesity (BMI ≥ 30 kg/m^2^) combined with low muscle strength (lowest sex-specific tertile) was associated with declines in walking speed and with increases in mobility disability, especially among persons younger than 80 years old.

Finally, Batsis and colleagues (14), analyzing 2,025 subjects aged 60 years and over with knee osteoarthritis over 4 years of follow-up, using BMI and knee extensor strength as the key exposure measures, found that obesity only (BMI ≥ 30 kg/m^2^), dynapenia only (lowest sex-specific tertile), and dynapenic obesity were associated with reduced gait speed at baseline among both sexes, with the worst performance observed among participants with dynapenic obesity. At baseline and over the follow-up period, dynapenic obese men were observed to have the worst performance in a 400-meter walking test. Unfortunately, the authors have not examined changes over time in ADL disability.

Rivera and colleagues (26) offer a conceptual model that delimits six domains that are necessary to mobility, and it is capable of explaining the decline in this function that can be used to explain disability in ADL. These six domains are as follows: central nervous system, peripheral nervous system, muscular system, osteoarticular system (bones and joints), perceptual system, and energy production. Despite the fact that the mechanism(s) whereby abdominal obesity contributes to a reduction in muscle strength has not yet been fully explained, some evidence that supports this relationship could contribute to the understanding of why dynapenic abdominal obese individuals showed worse trajectories of ADL disability by using Rivera’s conceptual model (27).

For example, body fat, especially abdominal fat, increases the expression of circulating cytokines as tumor necrosis factor-α, tumor necrosis factor-β, and interleukin-6, increasing muscle catabolic activity (28). In addition, TNF is also responsible for depressing the anabolic process and reduce the effect on myelination and repair of damaged axons through reduction of the effects mediated by the insulin-like growth factor-1 (29). Moreover, obesity, in particular central obesity, has been associated with intermuscular and intramuscular fat infiltration altering the muscular anatomy and impairing its function (30–34). Such modifications can undermine the functioning of the peripheral nervous system and muscular system. An impaired neuromuscular system could lead to difficulties in dealing with an overload in the osteoarticular system caused by abdominal obesity (35), increasing the progression of ADL disability.

This study provides some evidence to suggest that the combination of dynapenia and abdominal obesity is associated with a higher rate of increase in ADL disability among English older adults. Furthermore, our findings suggest that the use of models that fail to take the combination of these two conditions into account leads to an overestimation of the association between dynapenia only and abdominal obesity only on ADL disability trajectories.

Our study has several strengths and a number of potential limitations that need to be acknowledged. The first strength is the use of easy and standardized tools to detect the presence of abdominal obesity and dynapenia in clinical practice settings. Second, the present study was conducted on a large sample of community-dwelling older adults with a long period of follow-up. Third, we used mixed models in our analysis to accommodate the large number of confounding variables associated with ADL disability. Fourth, our analyses of ADL trajectories were run on the subset of participants without ADL disability at baseline (enabling us to minimize the influence of reverse causation). Fifth, our sensitivity analysis allowed us to show that the failure to account for the combination of dynapenia and abdominal obesity leads to an overestimation of their separate associations with the rate of change in ADL disability.

We acknowledge a number of limitations. Nonparticipation in the surveys over the follow-up period could be a source of bias. However, this type of bias is unavoidable in longitudinal studies of aging that only include community-dwelling older adults. Another source of bias relates to the generalizability of our findings. The ELSA participants who were excluded from our analytical sample were generally older, had lower handgrip strength, lower WC, lower scores on the tests of cognitive function, were more likely to report a sedentary lifestyle, were more likely to be current smokers, and were more likely to report the presence of a number of clinical conditions. Finally, the lack of information with regards to diet, age of onset of obesity, history of obesity, and the number of years of being overweight is also a limitation.

## Conclusions

Dynapenic abdominal obesity is an important risk factor for functional decline in older adults. Thus, our findings highlight the clinical importance of including abdominal obesity and dynapenia in the assessment of disability risk among older adults, particularly when both conditions are present in the same patient. Therefore, as abdominal obesity and dynapenia are potentially modifiable risk factors, our findings indicate potential paths for preventing or at least delaying the disability process in older adults.

## Funding

English Longitudinal Study of Ageing (ELSA) is funded by the National Institute on Aging/National Institutes of Health, USA (grant number 5R01AG017644-16) and by a consortium of the UK government departments coordinated by the Economic and Social Research Council (ESRC). Sao Paulo Research Foundation funds TSA (grant number 15/20294-4).

## Conflict of Interest

None reported.

## Supplementary Material

gly182_suppl_Supplementary_Table
