# Supplementary material for: Dynapenic Abdominal Obesity as a Risk Factor for Worse Trajectories of ADL Disability Among Older Adults: The ELSA Cohort Study
Source: J Gerontol A Biol Sci Med Sci. 2018 Aug 25;74(7):1112–8. doi: 10.1093/gerona/gly182 (PMC6580691; doi:10.1093/gerona/gly182)
Supplement: gly182_suppl_Supplementary_Table [file gly182_suppl_supplementary_table.docx]

**Supplemental Table 1.** Comparisons between included and excluded participants in ELSA at baseline.

|  | | **Included** | | **Excluded** |
| --- | --- | --- | --- | --- |
| **Sociodemographic characteristics** | |  | |  |
| Age, years (SD) | | 72.0 ± 0.1* | | 74.7 ± 0.3* |
| 60 – 69 Y.O. | | 53.8* | | 39.6* |
| 70 – 79 Y.O. | | 33.9* | | 37.4* |
| 80 or more Y.O. | | 12.3* | | 23.0* |
| Sex (female), (%) | | 54.0 | | 56.1 |
| Marital status (married), (%) | | 67.0* | | 55.6* |
| Household wealth, (%) | |  | |  |
| 5^th^ quintile (highest quintile) | | 24.3* | | 19.0* |
| 4^nd^ quintile | | 21.6* | | 17.8* |
| 3^th^ quintile | | 20.3* | | 18.2* |
| 2^th^ quintile | | 18.5* | | 20.7* |
| 1^st^ quintile (lowest quintile) | | 15.3* | | 24.3* |
| Schooling, (%) | |  | |  |
| Higher than A level | | 23.6* | | 18.3* |
| 0 level or equivalent | | 21.9* | | 15.2* |
| Less than 0 level or equivalent | | 54.5* | | 66.5* |
| **Behavioral characteristics** | |  | |  |
| Smoking, (%) | |  | |  |
| Non-smoker | | 37.6* | | 35.4* |
| Former-smoker | | 50.7* | | 48.8* |
| Current smoker | | 11.7* | | 15.8* |
| Alcohol intake, (%) | |  | |  |
| Non-drinkers or drank once a week | | 34.8* | | 31.0* |
| Drank frequently | | 39.1* | | 28.9* |
| Drank daily | | 17.2* | | 15.2* |
| Did not answer | | 8.9* | | 24.9* |
| Sedentary lifestyle, (%) | | 2.7* | | 6.9* |
| **Clinical Conditions** | |  | |  |
| Arterial hypertension (yes), (%) | | 18.5 | | 20.1 |
| Diabetes (yes), (%) | | 3.4 | | 4.8 |
| Cancer (yes), (%) | | 3.7 | | 3.9 |
| Lung disease (yes), (%) | | 12.4 | | 10.2 |
| Heart disease (yes), (%) | | 9.1 | | 10.3 |
| Stroke (yes), (%) | | 1.2* | | 2.4* |
| Osteoarthritis (yes), (%) | | 31.5 | | 32.8 |
| Falls (yes), (%) | | 26.9* | | 31.5* |
| Mean Memory Score, points (SD) | | 9.7 ± 0.1* | | 8.4 ± 0.1* |
| Depressive symptoms, (%) | | 10.3* | | 14.3* |
| Perception of vision, (%) | |  | |  |
| Good | | 89.2* | | 83.6* |
| Regular | | 8.6* | | 12.0* |
| Poor | | 2.2* | | 4.4* |
| Perception of hearing, (%) | |  | |  |
| Good | | 78.8 | | 76.6 |
| Regular | | 16.9 | | 17.6 |
| Poor | | 4.3 | | 5.8 |
| Hand grip strength, (kg) (SD) | | 30.2 ± 0.2* | | 27.7 ± 0.6* |
| Waist circumference, (cm) (SD) | | 94.8 ± 0.2* | | 92.8 ± 0.9* |
| Body Mass Index, kg/m^2^ (SD) | | 27.4 ± 0.1* | | 25.8 ± 0.4* |

Data are presented as percentages, means and standard deviation (SD). Wealth cut-points values: Highest quintile = more than £423k; 4th quintile = between £240k and £423k; 3rd quintile = between £137k and £240k; 2nd quintile = between £24k and £137k; Lowest quintile = less than £24k. * p-value < 0.05.
